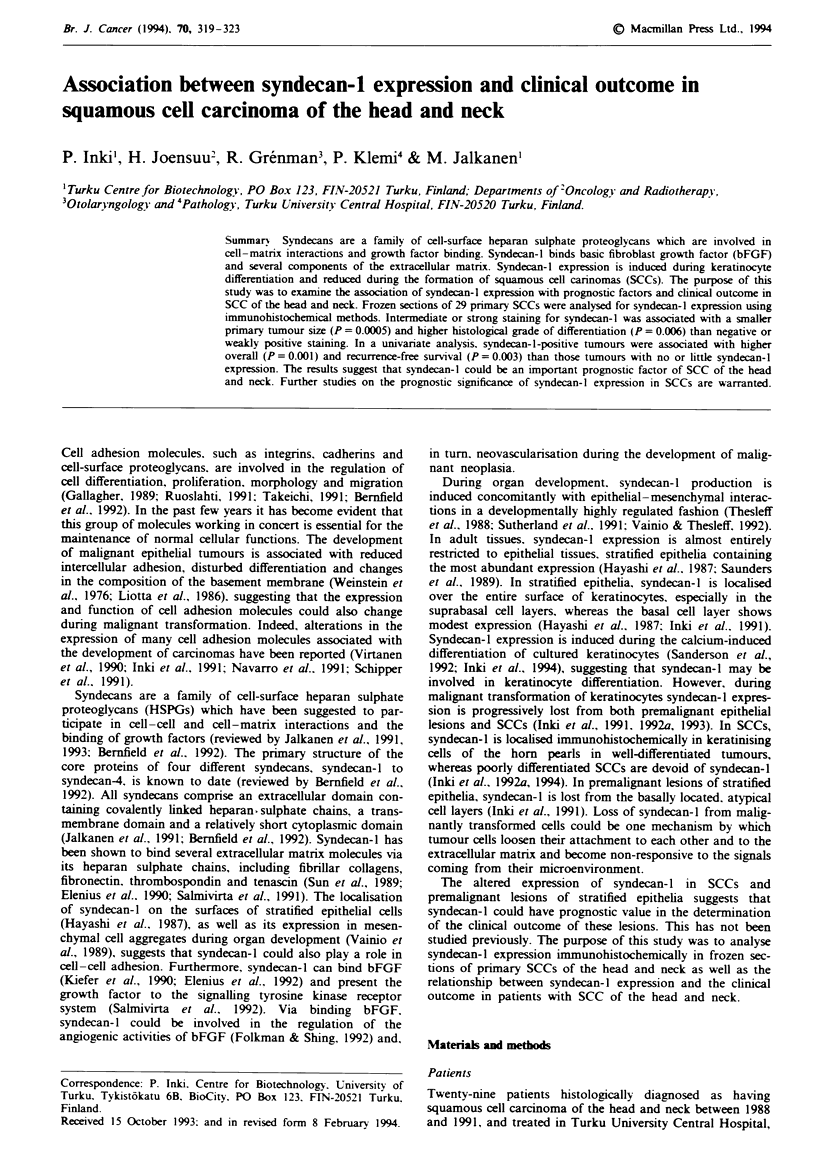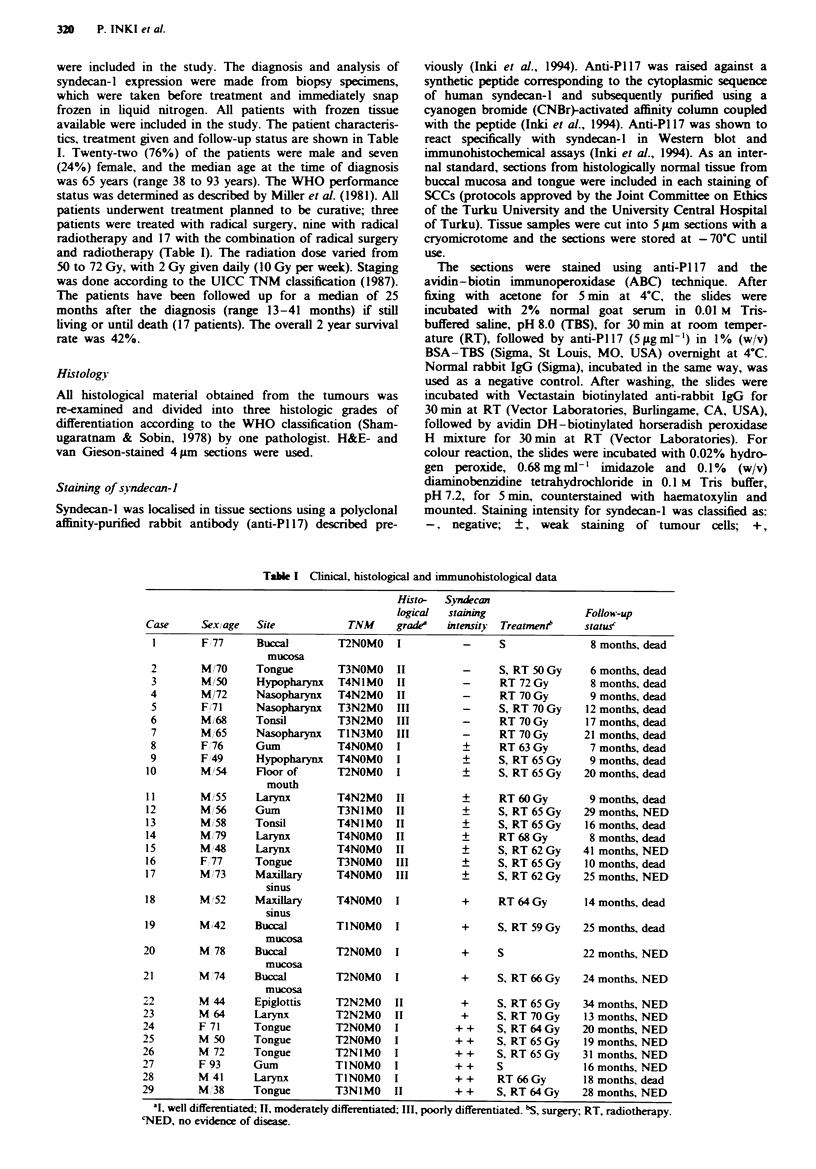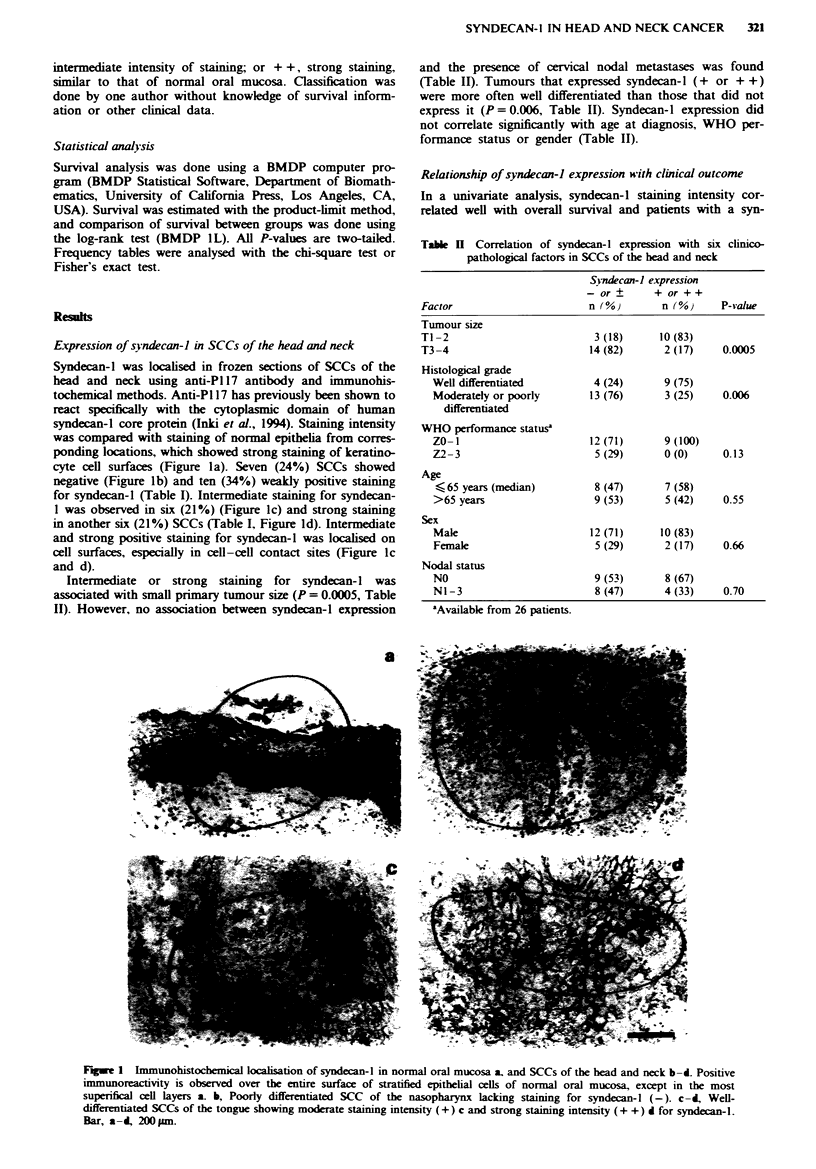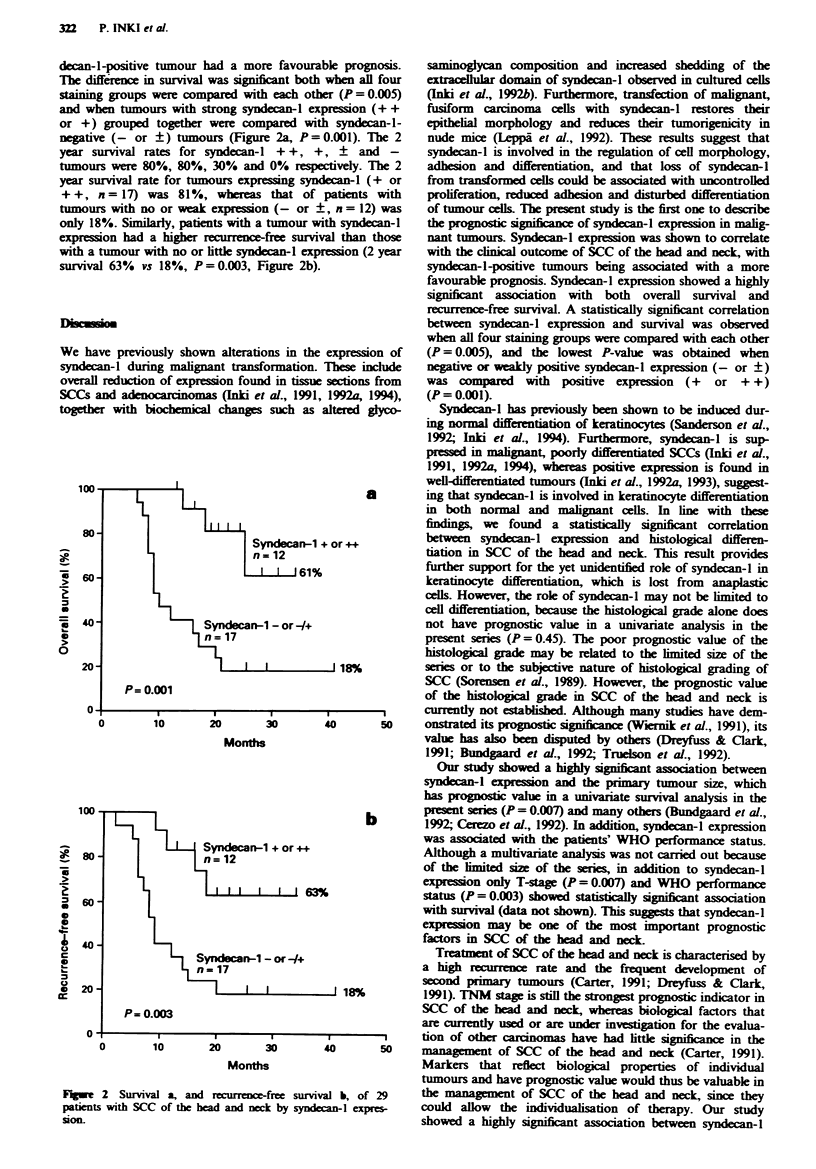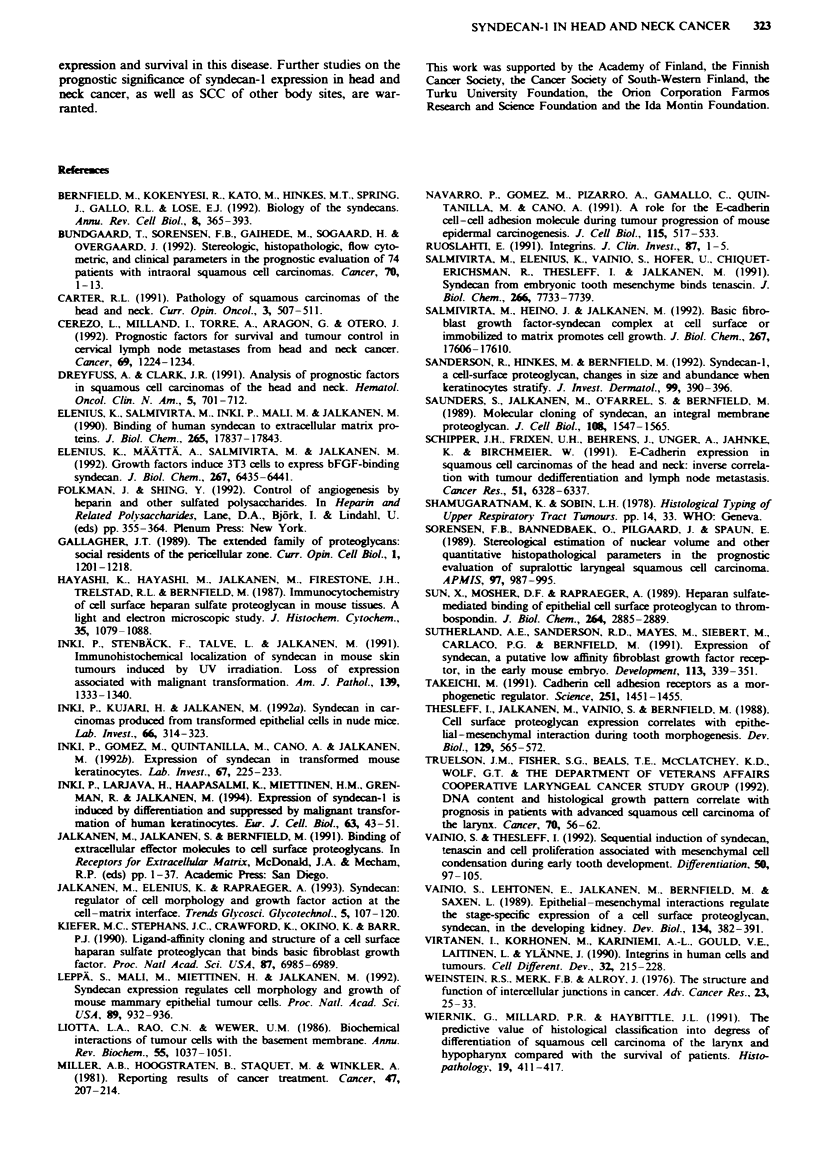# Association between syndecan-1 expression and clinical outcome in squamous cell carcinoma of the head and neck.

**DOI:** 10.1038/bjc.1994.300

**Published:** 1994-08

**Authors:** P. Inki, H. Joensuu, R. Grénman, P. Klemi, M. Jalkanen

**Affiliations:** Turku Centre for Biotechnology, Finland.

## Abstract

**Images:**


Br. J. Cancer (1994), 70, 319-323 © Macmillan Press Ltd., 1994

### Association between syndecan-1 expression and clinical outcome in squamous cell carcinoma of the head and neck

P. Inki[1], H. Joensuu[2], R. Grénman[3], P. Klemi[4] & M. Jalkanen[1]

[1]*Turku Centre for Biotechnology, PO Box 123, FIN-20521 Turku, Finland; Departments of* [2]*Oncology and Radiotherapy,* [3]*Otolaryngology and* [4]*Pathology, Turku University Central Hospital, FIN-20520 Turku, Finland.*

**Summary** Syndecans are a family of cell-surface heparan sulphate proteoglycans which are involved in cell-matrix interactions and growth factor binding. Syndecan-1 binds basic fibroblast growth factor (bFGF) and several components of the extracellular matrix. Syndecan-1 expression is induced during keratinocyte differentiation and reduced during the formation of squamous cell carinomas (SCCs). The purpose of this study was to examine the association of syndecan-1 expression with prognostic factors and clinical outcome in SCC of the head and neck. Frozen sections of 29 primary SCCs were analysed for syndecan-1 expression using immunohistochemical methods. Intermediate or strong staining for syndecan-1 was associated with a smaller primary tumour size ($P = 0.0005$) and higher histological grade of differentiation ($P = 0.006$) than negative or weakly positive staining. In a univariate analysis, syndecan-1-positive tumours were associated with higher overall ($P = 0.001$) and recurrence-free survival ($P = 0.003$) than those tumours with no or little syndecan-1 expression. The results suggest that syndecan-1 could be an important prognostic factor of SCC of the head and neck. Further studies on the prognostic significance of syndecan-1 expression in SCCs are warranted.

Cell adhesion molecules, such as integrins, cadherins and cell-surface proteoglycans, are involved in the regulation of cell differentiation, proliferation, morphology and migration (Gallagher, 1989; Ruoslahti, 1991; Takeichi, 1991; Bernfield *et al.*, 1992). In the past few years it has become evident that this group of molecules working in concert is essential for the maintenance of normal cellular functions. The development of malignant epithelial tumours is associated with reduced intercellular adhesion, disturbed differentiation and changes in the composition of the basement membrane (Weinstein *et al.*, 1976; Liotta *et al.*, 1986), suggesting that the expression and function of cell adhesion molecules could also change during malignant transformation. Indeed, alterations in the expression of many cell adhesion molecules associated with the development of carcinomas have been reported (Virtanen *et al.*, 1990; Inki *et al.*, 1991; Navarro *et al.*, 1991; Schipper *et al.*, 1991).

Syndecans are a family of cell-surface heparan sulphate proteoglycans (HSPGs) which have been suggested to participate in cell-cell and cell-matrix interactions and the binding of growth factors (reviewed by Jalkanen *et al.*, 1991, 1993; Bernfield *et al.*, 1992). The primary structure of the core proteins of four different syndecans, syndecan-1 to syndecan-4, is known to date (reviewed by Bernfield *et al.*, 1992). All syndecans comprise an extracellular domain containing covalently linked heparan sulphate chains, a transmembrane domain and a relatively short cytoplasmic domain (Jalkanen *et al.*, 1991; Bernfield *et al.*, 1992). Syndecan-1 has been shown to bind several extracellular matrix molecules via its heparan sulphate chains, including fibrillar collagens, fibronectin, thrombospondin and tenascin (Sun *et al.*, 1989; Elenius *et al.*, 1990; Salmivirta *et al.*, 1991). The localisation of syndecan-1 on the surfaces of stratified epithelial cells (Hayashi *et al.*, 1987), as well as its expression in mesenchymal cell aggregates during organ development (Vainio *et al.*, 1989), suggests that syndecan-1 could also play a role in cell-cell adhesion. Furthermore, syndecan-1 can bind bFGF (Kiefer *et al.*, 1990; Elenius *et al.*, 1992) and present the growth factor to the signalling tyrosine kinase receptor system (Salmivirta *et al.*, 1992). Via binding bFGF, syndecan-1 could be involved in the regulation of the angiogenic activities of bFGF (Folkman & Shing, 1992) and, in turn, neovascularisation during the development of malignant neoplasia.

During organ development, syndecan-1 production is induced concomitantly with epithelial-mesenchymal interactions in a developmentally highly regulated fashion (Thesleff *et al.*, 1988; Sutherland *et al.*, 1991; Vainio & Thesleff, 1992). In adult tissues, syndecan-1 expression is almost entirely restricted to epithelial tissues, stratified epithelia containing the most abundant expression (Hayashi *et al.*, 1987; Saunders *et al.*, 1989). In stratified epithelia, syndecan-1 is localised over the entire surface of keratinocytes, especially in the suprabasal cell layers, whereas the basal cell layer shows modest expression (Hayashi *et al.*, 1987; Inki *et al.*, 1991). Syndecan-1 expression is induced during the calcium-induced differentiation of cultured keratinocytes (Sanderson *et al.*, 1992; Inki *et al.*, 1994), suggesting that syndecan-1 may be involved in keratinocyte differentiation. However, during malignant transformation of keratinocytes syndecan-1 expression is progressively lost from both premalignant epithelial lesions and SCCs (Inki *et al.*, 1991, 1992*a*, 1993). In SCCs, syndecan-1 is localised immunohistochemically in keratinising cells of the horn pearls in well-differentiated tumours, whereas poorly differentiated SCCs are devoid of syndecan-1 (Inki *et al.*, 1992*a*, 1994). In premalignant lesions of stratified epithelia, syndecan-1 is lost from the basally located, atypical cell layers (Inki *et al.*, 1991). Loss of syndecan-1 from malignantly transformed cells could be one mechanism by which tumour cells loosen their attachment to each other and to the extracellular matrix and become non-responsive to the signals coming from their microenvironment.

The altered expression of syndecan-1 in SCCs and premalignant lesions of stratified epithelia suggests that syndecan-1 could have prognostic value in the determination of the clinical outcome of these lesions. This has not been studied previously. The purpose of this study was to analyse syndecan-1 expression immunohistochemically in frozen sections of primary SCCs of the head and neck as well as the relationship between syndecan-1 expression and the clinical outcome in patients with SCC of the head and neck.

### Materials and methods

### *Patients*

Twenty-nine patients histologically diagnosed as having squamous cell carcinoma of the head and neck between 1988 and 1991, and treated in Turku University Central Hospital,

Correspondence: P. Inki, Centre for Biotechnology, University of Turku, Tykistökatu 6B, BioCity, PO Box 123, FIN-20521 Turku, Finland.
Received 15 October 1993; and in revised form 8 February 1994.

were included in the study. The diagnosis and analysis of syndecan-1 expression were made from biopsy specimens, which were taken before treatment and immediately snap frozen in liquid nitrogen. All patients with frozen tissue available were included in the study. The patient characteristics, treatment given and follow-up status are shown in Table I. Twenty-two (76%) of the patients were male and seven (24%) female, and the median age at the time of diagnosis was 65 years (range 38 to 93 years). The WHO performance status was determined as described by Miller et al. (1981). All patients underwent treatment planned to be curative; three patients were treated with radical surgery, nine with radical radiotherapy and 17 with the combination of radical surgery and radiotherapy (Table I). The radiation dose varied from 50 to 72 Gy, with 2 Gy given daily (10 Gy per week). Staging was done according to the UICC TNM classification (1987). The patients have been followed up for a median of 25 months after the diagnosis (range 13–41 months) if still living or until death (17 patients). The overall 2 year survival rate was 42%.

*Histology*

All histological material obtained from the tumours was re-examined and divided into three histologic grades of differentiation according to the WHO classification (Shamugaratnam & Sobin, 1978) by one pathologist. H&E- and van Gieson-stained 4 μm sections were used.

*Staining of syndecan-1*

Syndecan-1 was localised in tissue sections using a polyclonal affinity-purified rabbit antibody (anti-P117) described previously (Inki et al., 1994). Anti-P117 was raised against a synthetic peptide corresponding to the cytoplasmic sequence of human syndecan-1 and subsequently purified using a cyanogen bromide (CNBr)-activated affinity column coupled with the peptide (Inki et al., 1994). Anti-P117 was shown to react specifically with syndecan-1 in Western blot and immunohistochemical assays (Inki et al., 1994). As an internal standard, sections from histologically normal tissue from buccal mucosa and tongue were included in each staining of SCCs (protocols approved by the Joint Committee on Ethics of the Turku University and the University Central Hospital of Turku). Tissue samples were cut into 5 μm sections with a cryomicrotome and the sections were stored at −70°C until use.

The sections were stained using anti-P117 and the avidin–biotin immunoperoxidase (ABC) technique. After fixing with acetone for 5 min at 4°C, the slides were incubated with 2% normal goat serum in 0.01 M Tris-buffered saline, pH 8.0 (TBS), for 30 min at room temperature (RT), followed by anti-P117 (5 $\mu g\ ml^{-1}$) in 1% (w/v) BSA–TBS (Sigma, St Louis, MO, USA) overnight at 4°C. Normal rabbit IgG (Sigma), incubated in the same way, was used as a negative control. After washing, the slides were incubated with Vectastain biotinylated anti-rabbit IgG for 30 min at RT (Vector Laboratories, Burlingame, CA, USA), followed by avidin DH-biotinylated horseradish peroxidase H mixture for 30 min at RT (Vector Laboratories). For colour reaction, the slides were incubated with 0.02% hydrogen peroxide, 0.68 $mg\ ml^{-1}$ imidazole and 0.1% (w/v) diaminobenzidine tetrahydrochloride in 0.1 M Tris buffer, pH 7.2, for 5 min, counterstained with haematoxylin and mounted. Staining intensity for syndecan-1 was classified as: −, negative; ±, weak staining of tumour cells; +,

**Table I** Clinical, histological and immunohistological data

| Case | Sex/age | Site | TNM | Histological grade[a] | Syndecan staining intensity | Treatment[b] | Follow-up status[c] |
|---|---|---|---|---|---|---|---|
| 1 | F/77 | Buccal mucosa | T2N0M0 | I | − | S | 8 months, dead |
| 2 | M/70 | Tongue | T3N0M0 | II | − | S, RT 50 Gy | 6 months, dead |
| 3 | M/50 | Hypopharynx | T4N1M0 | II | − | RT 72 Gy | 8 months, dead |
| 4 | M/72 | Nasopharynx | T4N2M0 | II | − | RT 70 Gy | 9 months, dead |
| 5 | F/71 | Nasopharynx | T3N2M0 | III | − | S, RT 70 Gy | 12 months, dead |
| 6 | M/68 | Tonsil | T3N2M0 | III | − | RT 70 Gy | 17 months, dead |
| 7 | M/65 | Nasopharynx | T1N3M0 | III | − | RT 70 Gy | 21 months, dead |
| 8 | F/76 | Gum | T4N0M0 | I | ± | RT 63 Gy | 7 months, dead |
| 9 | F/49 | Hypopharynx | T4N0M0 | I | ± | S, RT 65 Gy | 9 months, dead |
| 10 | M/54 | Floor of mouth | T2N0M0 | I | ± | S, RT 65 Gy | 20 months, dead |
| 11 | M/55 | Larynx | T4N2M0 | II | ± | RT 60 Gy | 9 months, dead |
| 12 | M/56 | Gum | T3N1M0 | II | ± | S, RT 65 Gy | 29 months, NED |
| 13 | M/58 | Tonsil | T4N1M0 | II | ± | S, RT 65 Gy | 16 months, dead |
| 14 | M/79 | Larynx | T4N0M0 | II | ± | RT 68 Gy | 8 months, dead |
| 15 | M/48 | Larynx | T4N0M0 | II | ± | S, RT 62 Gy | 41 months, NED |
| 16 | F/77 | Tongue | T3N0M0 | III | ± | S, RT 65 Gy | 10 months, dead |
| 17 | M/73 | Maxillary sinus | T4N0M0 | III | ± | S, RT 62 Gy | 25 months, NED |
| 18 | M/52 | Maxillary sinus | T4N0M0 | I | + | RT 64 Gy | 14 months, dead |
| 19 | M/42 | Buccal mucosa | T1N0M0 | I | + | S, RT 59 Gy | 25 months, dead |
| 20 | M/78 | Buccal mucosa | T2N0M0 | I | + | S | 22 months, NED |
| 21 | M/74 | Buccal mucosa | T2N0M0 | I | + | S, RT 66 Gy | 24 months, NED |
| 22 | M/44 | Epiglottis | T2N2M0 | II | + | S, RT 65 Gy | 34 months, NED |
| 23 | M/64 | Larynx | T2N2M0 | II | + | S, RT 70 Gy | 13 months, NED |
| 24 | F/71 | Tongue | T2N0M0 | I | ++ | S, RT 64 Gy | 20 months, NED |
| 25 | M/50 | Tongue | T2N0M0 | I | ++ | S, RT 65 Gy | 19 months, NED |
| 26 | M/72 | Tongue | T2N1M0 | I | ++ | S, RT 65 Gy | 31 months, NED |
| 27 | F/93 | Gum | T1N0M0 | I | ++ | S | 16 months, NED |
| 28 | M/41 | Larynx | T1N0M0 | I | ++ | RT 66 Gy | 18 months, dead |
| 29 | M/38 | Tongue | T3N1M0 | II | ++ | S, RT 64 Gy | 28 months, NED |

[a]I, well differentiated; II, moderately differentiated; III, poorly differentiated. [b]S, surgery; RT, radiotherapy. [c]NED, no evidence of disease.

intermediate intensity of staining; or + +, strong staining, similar to that of normal oral mucosa. Classification was done by one author without knowledge of survival information or other clinical data.

*Statistical analysis*

Survival analysis was done using a BMDP computer program (BMDP Statistical Software, Department of Biomathematics, University of California Press, Los Angeles, CA, USA). Survival was estimated with the product-limit method, and comparison of survival between groups was done using the log-rank test (BMDP 1L). All *P*-values are two-tailed. Frequency tables were analysed with the chi-square test or Fisher's exact test.

### Results

*Expression of syndecan-1 in SCCs of the head and neck*

Syndecan-1 was localised in frozen sections of SCCs of the head and neck using anti-P117 antibody and immunohistochemical methods. Anti-P117 has previously been shown to react specifically with the cytoplasmic domain of human syndecan-1 core protein (Inki *et al.*, 1994). Staining intensity was compared with staining of normal epithelia from corresponding locations, which showed strong staining of keratinocyte cell surfaces (Figure 1a). Seven (24%) SCCs showed negative (Figure 1b) and ten (34%) weakly positive staining for syndecan-1 (Table I). Intermediate staining for syndecan-1 was observed in six (21%) (Figure 1c) and strong staining in another six (21%) SCCs (Table I, Figure 1d). Intermediate and strong positive staining for syndecan-1 was localised on cell surfaces, especially in cell–cell contact sites (Figure 1c and d).

Intermediate or strong staining for syndecan-1 was associated with small primary tumour size ($P = 0.0005$, Table II). However, no association between syndecan-1 expression and the presence of cervical nodal metastases was found (Table II). Tumours that expressed syndecan-1 (+ or + +) were more often well differentiated than those that did not express it ($P = 0.006$, Table II). Syndecan-1 expression did not correlate significantly with age at diagnosis, WHO performance status or gender (Table II).

*Relationship of syndecan-1 expression with clinical outcome*

In a univariate analysis, syndecan-1 staining intensity correlated well with overall survival and patients with a syn-

**Table II** Correlation of syndecan-1 expression with six clinicopathological factors in SCCs of the head and neck

| Factor | *Syndecan-1 expression* – or ± n (%) | + or + + n (%) | P-*value* |
|---|---|---|---|
| Tumour size | | | |
| T1–2 | 3 (18) | 10 (83) | |
| T3–4 | 14 (82) | 2 (17) | 0.0005 |
| Histological grade | | | |
| Well differentiated | 4 (24) | 9 (75) | |
| Moderately or poorly differentiated | 13 (76) | 3 (25) | 0.006 |
| WHO performance status[a] | | | |
| Z0–1 | 12 (71) | 9 (100) | |
| Z2–3 | 5 (29) | 0 (0) | 0.13 |
| Age | | | |
| ≤65 years (median) | 8 (47) | 7 (58) | |
| >65 years | 9 (53) | 5 (42) | 0.55 |
| Sex | | | |
| Male | 12 (71) | 10 (83) | |
| Female | 5 (29) | 2 (17) | 0.66 |
| Nodal status | | | |
| N0 | 9 (53) | 8 (67) | |
| N1–3 | 8 (47) | 4 (33) | 0.70 |

[a]Available from 26 patients.

**Figure 1** Immunohistochemical localisation of syndecan-1 in normal oral mucosa **a**, and SCCs of the head and neck **b**–**d**. Positive immunoreactivity is observed over the entire surface of stratified epithelial cells of normal oral mucosa, except in the most superifical cell layers **a**. **b**, Poorly differentiated SCC of the nasopharynx lacking staining for syndecan-1 (–). **c**–**d**, Well-differentiated SCCs of the tongue showing moderate staining intensity (+) **c** and strong staining intensity (+ +) **d** for syndecan-1. Bar, **a**–**d**, 200 μm.

decan-1-positive tumour had a more favourable prognosis. The difference in survival was significant both when all four staining groups were compared with each other ($P = 0.005$) and when tumours with strong syndecan-1 expression (+ + or +) grouped together were compared with syndecan-1-negative (− or ±) tumours (Figure 2a, $P = 0.001$). The 2 year survival rates for syndecan-1 + +, +, ± and − tumours were 80%, 80%, 30% and 0% respectively. The 2 year survival rate for tumours expressing syndecan-1 (+ or + +, $n = 17$) was 81%, whereas that of patients with tumours with no or weak expression (− or ±, $n = 12$) was only 18%. Similarly, patients with a tumour with syndecan-1 expression had a higher recurrence-free survival than those with a tumour with no or little syndecan-1 expression (2 year survival 63% *vs* 18%, $P = 0.003$, Figure 2b).

### Discussion

We have previously shown alterations in the expression of syndecan-1 during malignant transformation. These include overall reduction of expression found in tissue sections from SCCs and adenocarcinomas (Inki *et al.*, 1991, 1992*a*, 1994), together with biochemical changes such as altered glycosaminoglycan composition and increased shedding of the extracellular domain of syndecan-1 observed in cultured cells (Inki *et al.*, 1992*b*). Furthermore, transfection of malignant, fusiform carcinoma cells with syndecan-1 restores their epithelial morphology and reduces their tumorigenicity in nude mice (Leppä *et al.*, 1992). These results suggest that syndecan-1 is involved in the regulation of cell morphology, adhesion and differentiation, and that loss of syndecan-1 from transformed cells could be associated with uncontrolled proliferation, reduced adhesion and disturbed differentiation of tumour cells. The present study is the first one to describe the prognostic significance of syndecan-1 expression in malignant tumours. Syndecan-1 expression was shown to correlate with the clinical outcome of SCC of the head and neck, with syndecan-1-positive tumours being associated with a more favourable prognosis. Syndecan-1 expression showed a highly significant association with both overall survival and recurrence-free survival. A statistically significant correlation between syndecan-1 expression and survival was observed when all four staining groups were compared with each other ($P = 0.005$), and the lowest $P$-value was obtained when negative or weakly positive syndecan-1 expression (− or ±) was compared with positive expression (+ or + +) ($P = 0.001$).

Syndecan-1 has previously been shown to be induced during normal differentiation of keratinocytes (Sanderson *et al.*, 1992; Inki *et al.*, 1994). Furthermore, syndecan-1 is suppressed in malignant, poorly differentiated SCCs (Inki *et al.*, 1991, 1992*a*, 1994), whereas positive expression is found in well-differentiated tumours (Inki *et al.*, 1992*a*, 1993), suggesting that syndecan-1 is involved in keratinocyte differentiation in both normal and malignant cells. In line with these findings, we found a statistically significant correlation between syndecan-1 expression and histological differentiation in SCC of the head and neck. This result provides further support for the yet unidentified role of syndecan-1 in keratinocyte differentiation, which is lost from anaplastic cells. However, the role of syndecan-1 may not be limited to cell differentiation, because the histological grade alone does not have prognostic value in a univariate analysis in the present series ($P = 0.45$). The poor prognostic value of the histological grade may be related to the limited size of the series or to the subjective nature of histological grading of SCC (Sorensen *et al.*, 1989). However, the prognostic value of the histological grade in SCC of the head and neck is currently not established. Although many studies have demonstrated its prognostic significance (Wiernik *et al.*, 1991), its value has also been disputed by others (Dreyfuss & Clark, 1991; Bundgaard *et al.*, 1992; Truelson *et al.*, 1992).

Our study showed a highly significant association between syndecan-1 expression and the primary tumour size, which has prognostic value in a univariate survival analysis in the present series ($P = 0.007$) and many others (Bundgaard *et al.*, 1992; Cerezo *et al.*, 1992). In addition, syndecan-1 expression was associated with the patients' WHO performance status. Although a multivariate analysis was not carried out because of the limited size of the series, in addition to syndecan-1 expression only T-stage ($P = 0.007$) and WHO performance status ($P = 0.003$) showed statistically significant association with survival (data not shown). This suggests that syndecan-1 expression may be one of the most important prognostic factors in SCC of the head and neck.

Treatment of SCC of the head and neck is characterised by a high recurrence rate and the frequent development of second primary tumours (Carter, 1991; Dreyfuss & Clark, 1991). TNM stage is still the strongest prognostic indicator in SCC of the head and neck, whereas biological factors that are currently used or are under investigation for the evaluation of other carcinomas have had little significance in the management of SCC of the head and neck (Carter, 1991). Markers that reflect biological properties of individual tumours and have prognostic value would thus be valuable in the management of SCC of the head and neck, since they could allow the individualisation of therapy. Our study showed a highly significant association between syndecan-1

**Figure 2** Survival **a**, and recurrence-free survival **b**, of 29 patients with SCC of the head and neck by syndecan-1 expression.

expression and survival in this disease. Further studies on the prognostic significance of syndecan-1 expression in head and neck cancer, as well as SCC of other body sites, are warranted.

This work was supported by the Academy of Finland, the Finnish Cancer Society, the Cancer Society of South-Western Finland, the Turku University Foundation, the Orion Corporation Farmos Research and Science Foundation and the Ida Montin Foundation.

### References

BERNFIELD, M., KOKENYESI, R., KATO, M., HINKES, M.T., SPRING, J., GALLO, R.L. & LOSE, E.J. (1992). Biology of the syndecans. *Annu. Rev. Cell Biol.*, **8**, 365-393.

BUNDGAARD, T., SORENSEN, F.B., GAIHEDE, M., SOGAARD, H. & OVERGAARD, J. (1992). Stereologic, histopathologic, flow cytometric, and clinical parameters in the prognostic evaluation of 74 patients with intraoral squamous cell carcinomas. *Cancer*, **70**, 1-13.

CARTER, R.L. (1991). Pathology of squamous carcinomas of the head and neck. *Curr. Opin. Oncol.*, **3**, 507-511.

CEREZO, L., MILLAND, I., TORRE, A., ARAGON, G. & OTERO, J. (1992). Prognostic factors for survival and tumour control in cervical lymph node metastases from head and neck cancer. *Cancer*, **69**, 1224-1234.

DREYFUSS, A. & CLARK, J.R. (1991). Analysis of prognostic factors in squamous cell carcinomas of the head and neck. *Hematol. Oncol. Clin. N. Am.*, **5**, 701-712.

ELENIUS, K., SALMIVIRTA, M., INKI, P., MALI, M. & JALKANEN, M. (1990). Binding of human syndecan to extracellular matrix proteins. *J. Biol. Chem.*, **265**, 17837-17843.

ELENIUS, K., MÄÄTTÄ, A., SALMIVIRTA, M. & JALKANEN, M. (1992). Growth factors induce 3T3 cells to express bFGF-binding syndecan. *J. Biol. Chem.*, **267**, 6435-6441.

FOLKMAN, J. & SHING, Y. (1992). Control of angiogenesis by heparin and other sulfated polysaccharides. In *Heparin and Related Polysaccharides*, Lane, D.A., Björk, I. & Lindahl, U. (eds) pp. 355-364. Plenum Press: New York.

GALLAGHER, J.T. (1989). The extended family of proteoglycans: social residents of the pericellular zone. *Curr. Opin. Cell Biol.*, **1**, 1201-1218.

HAYASHI, K., HAYASHI, M., JALKANEN, M., FIRESTONE, J.H., TRELSTAD, R.L. & BERNFIELD, M. (1987). Immunocytochemistry of cell surface heparan sulfate proteoglycan in mouse tissues. A light and electron microscopic study. *J. Histochem. Cytochem.*, **35**, 1079-1088.

INKI, P., STENBÄCK, F., TALVE, L. & JALKANEN, M. (1991). Immunohistochemical localization of syndecan in mouse skin tumours induced by UV irradiation. Loss of expression associated with malignant transformation. *Am. J. Pathol.*, **139**, 1333-1340.

INKI, P., KUJARI, H. & JALKANEN, M. (1992*a*). Syndecan in carcinomas produced from transformed epithelial cells in nude mice. *Lab. Invest.*, **66**, 314-323.

INKI, P., GOMEZ, M., QUINTANILLA, M., CANO, A. & JALKANEN, M. (1992*b*). Expression of syndecan in transformed mouse keratinocytes. *Lab. Invest.*, **67**, 225-233.

INKI, P., LARJAVA, H., HAAPASALMI, K., MIETTINEN, H.M., GRENMAN, R. & JALKANEN, M. (1994). Expression of syndecan-1 is induced by differentiation and suppressed by malignant transformation of human keratinocytes. *Eur. J. Cell. Biol.*, **63**, 43-51.

JALKANEN, M., JALKANEN, S. & BERNFIELD, M. (1991). Binding of extracellular effector molecules to cell surface proteoglycans. In *Receptors for Extracellular Matrix*, McDonald, J.A. & Mecham, R.P. (eds) pp. 1-37. Academic Press: San Diego.

JALKANEN, M., ELENIUS, K. & RAPRAEGER, A. (1993). Syndecan: regulator of cell morphology and growth factor action at the cell-matrix interface. *Trends Glycosci. Glycotechnol.*, **5**, 107-120.

KIEFER, M.C., STEPHANS, J.C., CRAWFORD, K., OKINO, K. & BARR, P.J. (1990). Ligand-affinity cloning and structure of a cell surface haparan sulfate proteoglycan that binds basic fibroblast growth factor. *Proc. Natl Acad. Sci. USA*, **87**, 6985-6989.

LEPPÄ, S., MALI, M., MIETTINEN, H. & JALKANEN, M. (1992). Syndecan expression regulates cell morphology and growth of mouse mammary epithelial tumour cells. *Proc. Natl. Acad. Sci. USA*, **89**, 932-936.

LIOTTA, L.A., RAO, C.N. & WEWER, U.M. (1986). Biochemical interactions of tumour cells with the basement membrane. *Annu. Rev. Biochem.*, **55**, 1037-1051.

MILLER, A.B., HOOGSTRATEN, B., STAQUET, M. & WINKLER, A. (1981). Reporting results of cancer treatment. *Cancer*, **47**, 207-214.

NAVARRO, P., GOMEZ, M., PIZARRO, A., GAMALLO, C., QUINTANILLA, M. & CANO, A. (1991). A role for the E-cadherin cell-cell adhesion molecule during tumour progression of mouse epidermal carcinogenesis. *J. Cell Biol.*, **115**, 517-533.

RUOSLAHTI, E. (1991). Integrins. *J. Clin. Invest.*, **87**, 1-5.

SALMIVIRTA, M., ELENIUS, K., VAINIO, S., HOFER, U., CHIQUET-ERICHSMAN, R., THESLEFF, I. & JALKANEN, M. (1991). Syndecan from embryonic tooth mesenchyme binds tenascin. *J. Biol. Chem.*, **266**, 7733-7739.

SALMIVIRTA, M., HEINO, J. & JALKANEN, M. (1992). Basic fibroblast growth factor-syndecan complex at cell surface or immobilized to matrix promotes cell growth. *J. Biol. Chem.*, **267**, 17606-17610.

SANDERSON, R., HINKES, M. & BERNFIELD, M. (1992). Syndecan-1, a cell-surface proteoglycan, changes in size and abundance when keratinocytes stratify. *J. Invest. Dermatol.*, **99**, 390-396.

SAUNDERS, S., JALKANEN, M., O'FARREL, S. & BERNFIELD, M. (1989). Molecular cloning of syndecan, an integral membrane proteoglycan. *J. Cell Biol.*, **108**, 1547-1565.

SCHIPPER, J.H., FRIXEN, U.H., BEHRENS, J., UNGER, A., JAHNKE, K. & BIRCHMEIER, W. (1991). E-Cadherin expression in squamous cell carcinomas of the head and neck: inverse correlation with tumour dedifferentiation and lymph node metastasis. *Cancer Res.*, **51**, 6328-6337.

SHAMUGARATNAM, K. & SOBIN, L.H. (1978). *Histological Typing of Upper Respiratory Tract Tumours*. pp. 14, 33. WHO: Geneva.

SORENSEN, F.B., BANNEDBAEK, O., PILGAARD, J. & SPAUN, E. (1989). Stereological estimation of nuclear volume and other quantitative histopathological parameters in the prognostic evaluation of supralottic laryngeal squamous cell carcinoma. *APMIS*, **97**, 987-995.

SUN, X., MOSHER, D.F. & RAPRAEGER, A. (1989). Heparan sulfate-mediated binding of epithelial cell surface proteoglycan to thrombospondin. *J. Biol. Chem.*, **264**, 2885-2889.

SUTHERLAND, A.E., SANDERSON, R.D., MAYES, M., SIEBERT, M., CARLACO, P.G. & BERNFIELD, M. (1991). Expression of syndecan, a putative low affinity fibroblast growth factor receptor, in the early mouse embryo. *Development*, **113**, 339-351.

TAKEICHI, M. (1991). Cadherin cell adhesion receptors as a morphogenetic regulator. *Science*, **251**, 1451-1455.

THESLEFF, I., JALKANEN, M., VAINIO, S. & BERNFIELD, M. (1988). Cell surface proteoglycan expression correlates with epithelial-mesenchymal interaction during tooth morphogenesis. *Dev. Biol.*, **129**, 565-572.

TRUELSON, J.M., FISHER, S.G., BEALS, T.E., MCCLATCHEY, K.D., WOLF, G.T. & THE DEPARTMENT OF VETERANS AFFAIRS COOPERATIVE LARYNGEAL CANCER STUDY GROUP (1992). DNA content and histological growth pattern correlate with prognosis in patients with advanced squamous cell carcinoma of the larynx. *Cancer*, **70**, 56-62.

VAINIO, S. & THESLEFF, I. (1992). Sequential induction of syndecan, tenascin and cell proliferation associated with mesenchymal cell condensation during early tooth development. *Differentiation*, **50**, 97-105.

VAINIO, S., LEHTONEN, E., JALKANEN, M., BERNFIELD, M. & SAXEN, L. (1989). Epithelial-mesenchymal interactions regulate the stage-specific expression of a cell surface proteoglycan, syndecan, in the developing kidney. *Dev. Biol.*, **134**, 382-391.

VIRTANEN, I., KORHONEN, M., KARINIEMI, A.-L., GOULD, V.E., LAITINEN, L. & YLÄNNE, J. (1990). Integrins in human cells and tumours. *Cell Different. Dev.*, **32**, 215-228.

WEINSTEIN, R.S., MERK, F.B. & ALROY, J. (1976). The structure and function of intercellular junctions in cancer. *Adv. Cancer Res.*, **23**, 25-33.

WIERNIK, G., MILLARD, P.R. & HAYBITTLE, J.L. (1991). The predictive value of histological classification into degress of differentiation of squamous cell carcinoma of the larynx and hypopharynx compared with the survival of patients. *Histopathology*, **19**, 411-417.